# Multivariate analysis of factors associated with *Schistosoma mansoni* and hookworm infection among primary school children in rural Bahir Dar, Northwest Ethiopia

**DOI:** 10.1186/s40794-018-0064-6

**Published:** 2018-06-01

**Authors:** Tadesse Hailu, Megbaru Alemu, Bayeh Abera, Wondemagegn Mulu, Endalew Yizengaw, Ashenafi Genanew, Fetlework Bereded

**Affiliations:** 1 Department of Medical Laboratory Science, Bahir Dar University, Bahir Dar, Ethiopia; 2 Department of Pharmacy, Bahir Dar University, Bahir Dar, Ethiopia

**Keywords:** Helminth, Hookworm, Schistosoma, Sebatamit, Bahir Dar

## Abstract

**Background:**

Soil-transmitted helminths and *Schistosoma mansoni* infections are the major causes of morbidity and mortality in Sub-Saharan countries. The highest burden of the disease resides in school-age children. Poor water sanitation and hygiene are believed to be the major contributing factors for the high prevalence. Therefore, the goal of this study was to determine the prevalence of intestinal parasite infections in rural Bahir Dar, Northwest Ethiopia.

**Methods:**

A cross-sectional study was conducted from April 2017–June 2017 among 409 randomly selected primary school children. A structured questionnaire was used to obtain socio-demographic information and determinant factors through interviewing the students. Stool examination was done by Ritchie’s concentration method. The data were entered and analyzed using SPSS version 22. Prevalence of helminthic infections was calculated using descriptive statistics. The association between helminthic infection and determinant factors was determined by Bavarian regression. The confounding effect was checked by multivariate regression at 95% confidence interval. Any association was significant when the *p*-value was < 0.05.

**Result:**

The overall prevalence of intestinal parasite infection was 47.2%.(193/409).. The prevalence of Hookworm species and *Schistosoma mansoni* was 31.1 and 8.0%, respectively. Co-infection of Hookworm species with *Schistosoma mansoni* was 5.1% (21/409). The highest prevalence of *Schistosoma mansoni* was recorded for boys (21%), older children (21.4%) and rural children (17.6%) (*P* < 0.05). *Schistosoma mansoni* infection was also higher among children whose household drinking water was sourced from streams/rivers (P < 0.05). The multivariate analysis showed lower odds of *Schistosoma mansoni* infection for those with no history of bathing (AOR = 3.7, 95% CI: 1.1–12.2; *P* = 0.034), washing clothes/utensils (AOR = 3.4; 95% CI: 1.2–9.7; *P* = 0.022), swimming (AOR = 2.8, 95% CI: 1.2–6.9; *P* = 0.023), and irrigation (AOR = 2.8, 95% CI: 1.3–6.0; *P* = 0.01). Significantly, higher odds of Hookworm infection was recorded for older children (AOR = 2.3, 95% CI: 1.08–4.89; *P* = 0.029), boys (AOR = 1.9, 95% CI: 1.12–3.24; *P* = 0.018), and rural children (AOR = 1.8, 95% CI: 1.04–3.0; *P* = 0.037). Regular shoe wearing (AOR = 0.29, 95% CI: 0.16–0.50; *P* = 0.00) is protective for hookworm infection. Higher odds of hookworm infection was also recorded for schoolchildren who had the habit of eating raw vegetables (AOR = 1.2 95% CI: 1.1–1.7 *P* = 0.011).

**Conclusion:**

Hookworm infection and schistosomiasis are prevalent in the school children in rural Bahir Dar in Northwest Ethiopia. Various activities and behaviors of the children were strongly associated with helminthic infection. Hence health education should be delivered regularly to minimize/avoid the risky behaviors and water-based activities. Deworming programs should also be implemented on a regular basis.

## Background

Soil-transmitted helminthiasis and schistosomiasis are the most common parasitic infections especially in developing countries [1]. Nearly, 1.5 billion people (24% of the world’s population) are infected with soil-transmitted helminths.More than 267 million preschool-age children and over 568 million school-age children live in areas where these parasites are intensively transmitted. [2]. Based on the World Health Organization (WHO) estimation, there are 700–900 million hookworm and 200 million Schistosoma infections worldwide [3]. They are the major causes of morbidity and mortality in the Sub-Saharan countries [3, 4]. The disease burden and the public health importance of these parasites are still concerns in developing countries like Ethiopia [5].

Poverty, poor living conditions, personal and environmental hygiene, sanitation and water supply facilities are some of the contributing factors for the high prevalence of soil-transmitted helminths and *Schistosoma mansoni* [6]. Moreover, the high prevalence of schistosomiasis is mainly associated with poor water management system during irrigation, close contact habit to freshwater bodies, open defecation and wide distribution of the snail intermediate host [7].

Infections by soil-transmitted helminths and *Schistosoma* species lead to malnutrition, iron deficiency anemia, stunted growth and increased vulnerability to other infections [8] and low educational achievement in school children [9].Current control programs for helminths infection focused on reducing infection intensity and transmission potential, primarily to reduce morbidity and avoid mortality associated with the disease [10].

Despite a range of prevention and control efforts of helminthic infections, the prevalence of soil-transmitted helminths and schistosome infections is still high in several localities of Ethiopia. There is also a lack of information on the factors of *S. mansoni* and soil-transmitted helminths infections in the study area. Therefore, the aim of this study was to assess the magnitude of helminth infections and associated risk factors so that to design effective prevention and control measures.

## Materials and methods

### Study design, area and period

A cross-sectional study, aimed at determining the prevalence of intestinal parasite infections and determinant factors among school children was conducted in rural Bahir Dar, Amhara Regional National State, Northwest Ethiopia from April–June 2017.

The altitude of the area is between 648 and 1300 m above sea level. The annual temperature and rainfall of the study area are 18–22 °C and 1419 mm, respectively. There is a small-scale irrigation on Andasa and Abay rivers in the study area.

In this particular study, Sebatamit primary school students age ranging from 7 to 14 years and willing to participate in the study were included. A total of 409 students from grade 1–7 were recruited to the study. Systematic random sampling technique was used to select the classes in the school and the students in each class. All Sebatamit primary school students aged 7–14 years and signed the assent form and parents signed written consent forms were included.

### Data collection

A structured questionnaire was used to obtain socio-demographic information, present and past history of the participants and environmental related factors by interviewing the students. The questionnaire was filled by nurses. The data collectors were regularly supervised within ten participants’ variation by the principal investigator.

Freshly passed stool specimens were collected using a clean plastic cup at the school. The stool cups were labeled based on their section and role number. The laboratory professionals took part in all processes of stool collection, transportation, and examination. The stool samples were processed for microscopic examination by Ritchie’s concentration method. The stool examination was done in Bahir Dar University, College of Medicine and Health Sciences, Department of Microbiology, Immunology, and Parasitology teaching laboratory.

#### Ritchie’s concentration method

About 2.5 ml of formalin and 1 ml of ethyl acetate was in the sample collection tube. Then, 0.5 g of stool sample was put inside the sample collection tube and left to stand for a minute. Both pieces of the device were screwed until it is well closed and centrifuged at 1000 rpm for 3 min. The sample collection tube and filtration concentration unit were discarded. The supernatant was removed from the conical tube and a small amount of sediment was put on a glass slide for examination at 10× & 40× objectives.

### Data quality assurance

To ensure reliable data collection, training of nurses and laboratory technicians on data collection and explanation about the study were given before sample collection. The data collection, application of standard procedures, and accuracy of test results were supervised by the principal investigator. Close follow up by the principal investigator within ten patients’ variation during data collection process was done. Specimens were cross-checked by the principal investigator to increase the accuracy of laboratory results.

To eliminate observer bias, the smears were examined independently with experienced laboratory technologists and 10% of the total slides will be randomly selected and read by the principal investigator. The results of their observation were recorded for later comparison on separate sheets. A quality control was done by repeating all discordant results.

### Data analysis

Data were entered and analyzed using SPSS version 22 statistical software. Overall magnitude, of parasitic infection, was calculated using descriptive statistics. The strength of association between parasites infections and determinant factors was calculated by binary logistic statistics. Those variables with *P* < 0.2 in the binary logistic regression were taken to multiple regression analysis and the AOR was calculated to control potential confounders. *P*-values less than 0.05 were taken statistical significant.

## Results

The overall prevalence of single, double and triple intestinal parasite infection was 47.2, 10.0 and 0.98%, respectively. Hookworms (31.7%) were the predominant species identified followed by *Schistosoma mansoni* (8.0%), and *Entamoeba histolytica* (5.4%) (Table 1). The highest prevalence of *S.mansoni* was recorded for boys (21%), older children (21.4%) and rural children (17.6%) (*P* < 0.05). *S.mansoni* infection was also higher among children whose family drinking water was sourced from streams (P < 0.05). The odds of infection was higher in this group (AOR = 0.34, 95% CI: 0.162–0.707; *P* = 0.004) (Table 2).

**Table 1:** Prevalence of intestinal parasites among primary schoolchildren in rural Bahir Dar, Northwest Ethiopia, 2017

Parasite species	No infected	Percent (*n* = 409)
Single infection		
Hookworm *Spp*	130	31.7
*Schistosoma mansoni*	33	8.0
*Entamoeba histolytica*	22	5.4
*Ascaris lumbricoides*	5	1.2
*Strongyloides stercolaris*	1	0.2
*Taenia* spp	1	0.2
*Giardia lamblia*	1	0.2
Double infection		
*S. mansoni* + Hookworm *spp*	17	4.2
Hookworm *spp* + *E. histolytica*	16	3.9
Hookworm + *Giardia lamblia*	3	0.7
*E. histolytica* + *Giardia lamblia*	2	0.5
*A.lumbricoides* + *E. histolytica*	1	0.2
*S. mansoni* + *E. histolytica*	1	0.2
*S. mansoni* + *A. lumbricoides*	1	0.2
Triple infection		
*S. mansoni* + Hookworm *spp* + *E. histolytica*	1	0.2
*S. mansoni* + Hookworm *spp* + *G. lamblia*	2	0.5
*S. mansoni* + Hookworm *spp* + *S. stercolaris*	1	0.2
Negative	172	42.0

**Table 2:** The prevalence and factors associated with *Schistosoma mansoni* among primary schoolchildren in rural Bahir Dar, Northwest Ethiopia, 2017

Variables		*Schistosma mansoni* infection			P-value	AOR (95% CI)
		Positive *n (%)*	Negative *n (%)*	Total (n = 409)		
Age						
7–10		28 (10.1)	250 (89.9)	278 (68.0)	0.002	2.1 (1.1–4.2)
11–14		28 (21.4)	103 (78.6)	131 (32.0)		
Sex						
Male		39 (21.0)	147 (79.0)	186 (45.5)	0.002	2.9 (1.5–5.7)
Female		17 (7.6)	206 (92.4)	223 (54.5)		
Residence						
Urban		41 (17.6)	192 (82.4)	223 (57.0)	0.002	0.301 (0.14–0.64)
Rural		15 (8.5)	161 (91.5)	176 (43.0)		
Water source						
Pipe		15 (7.5)	184 (92.5)	199 (48.7)	0.004	3.0 (1.41–6.2)
Stream		41 (19.5)	169 (80.5)	210 (51.3)		
Bathing						
Yes		52 (18.3)	232 (81.7)	284 (69.4)	0.034	3.7 (1.1–12.2)
No		4 (3.2)	121 (96.8)	125 (30.6)		
Washing clothes						
Yes		51 (19.2)	215 (80.8)	266 (65.0)	0.022	3.4 (1.2–9.7)
No		5 (3.5)	138 (96.5)	143 (35.0)		
Swimming						
Yes		49 (20.8)	187 (79.2)	236 (57.7)	0.023	2.8 (1.2–6.9)
No		7 (4.0)	166 (96.0)	173 (42.3)		
Irrigation						
Yes		45 (19.7)	183 (80.3)	228 (55.7)	0.010	2.8 (1.3–6.0)
No		11 (6.1)	170 (93.9)	181 (44.3)		
Total	Positive	56 (13.7)	353 (86.3)	409 (100)		

Domestic activities accounted for the majority of reported water contacts. The majority (69.4%) of subjects interviewed reported that they bathed in the nearby stream. Similarly, about 65% of the respondents encountered water contact for washing clothes. Besides, 57.7% of the school children frequently practiced swimming. About half of respondents reported that the water for drinking and cooking was collected from streams. School children who frequently encounter contact with stream/river water during bathing (AOR = 3.7, 95% CI: 1.1–12.2; *P* = 0.034), washing clothes (AOR = 3.4, 95% CI: 1.2–9.7; *P* = 0.022), swimming (AOR = 2.8, 95% CI:1.2–6.9; *P* = 0.023) and irrigation (AOR = 2.8, 95% CI:1.3–6.0; *P* = 0.01) had higher odds of *S. mansoni* infection (Table 2).

The overall prevalence of hookworm infection was 41.1%. Significantly higher proportions of Hookworm infection were recorded for older children (AOR = 2.3, 95% CI: 1.08–4.89; *P* = 0.029), boys (AOR = 1.9, 95% CI: 1.12–3.24; *P* = 0.018), rural children (AOR = 1.8, 95% CI: 1.04–3.0; *P* = 0.037). Regular shoe wearing (AOR = 0.29, 95% CI: 0.16–0.50; *P* = 0.00) was protective for hookworm infection. Higher odds of hookworm infection was also recorded among schoolchildren who did not have the habit of regular latrine usage (55.8%), those practicing geophagy (50.6%) and eat raw vegetables (48.2%) (Table 3).

**Table 3:** The prevalence and factors associated with Hookworm infection among primary schoolchildren in rural Bahir Dar, Northwest Ethiopia, 2017

Variables		Hookworm infection			P-value	AOR (95% CI)
		Positive *n* (%)	Negative *n* (%)	Total (*n* = 409)		
Age (Yrs)	7–9	154 (44.5)	192 (55.5)	346 (84.6)	.029	2.3 (1.09–4.89)
	10–14	14 (22.2)	49 (77.8)	63 (15.4)		
Sex	Male	78 (41.9)	108 (58.1)	186 (45.5)	.018	1.9 (1.12–3.24)
	Female	90 (40.4)	133 (59.6)	223 (54.5)		
Residence	Urban	74 (31.8)	159 (68.2)	223 (57.0)	.037	1.8 (1.04–3.01)
	Rural	94 (53.4)	82 (46.6)	176 (43.0)		
Hand wash before meal	Yes	128 (36.1)	227 (63.9)	355 (86.8)	.010	2.9 (1.29–6.65)
	No	40 (74.1)	14 (25.9)	54 (13.2)		
Regular latrine use	Yes	28 (17.7)	130 (82.3)	158 (28.6)	.012	2.2 (1.19–4.18)
	No	140 (55.8)	111 (44.2)	251 (61.4)		
Frequency of shoe wearing	Always	44 (10.8)	163 (67.6)	1 (0.2)	.000	0.3 (0.16–0.50)
	Sometimes	124 (30.3)	78 (32.4)	408 (99.8)		
Geophagy	Yes	44 (50.6)	43 (49.4)	87 (21.3)	.052	1.6 (1.0–2.6)
	No	124 (38.5)	198 (61.5)	322 (78.7)		
Irrigation	Yes	90 (39.5)	138 (60.5)	228 (55.7)	.050	1.61 (1.00–2.60)
	No	78 (43.1)	103 (56.9)	181 (44.3)		
Eating raw vegetables	Yes	137 (48.2)	147 (51.8)	284 (69.4)	.011	1.2 (1.1–1.7)
	No	3 1(24.8)	94 (75.2)	125 (30.6)		
Total	Positive	168 (41.1)	241 (58.9)			

## Discussion

Epidemiological study on the prevalence and distribution of helminthic species in different localities is a primary objective to identify high-risk communities and formulate appropriate intervention [11]. Assessing factors associated with the infection of parasites enables determining priorities for public health measures to prevent parasitic infections [12].

The overall prevalence of *S. mansoni* was 8.0% in our study and it was lower than other studies conducted among school children in different parts of Ethiopia which reported prevalence greater than 30% [13–18]. This lower prevalence might be attributed to the sporadic deworming programs conducted by Amhara Regional Health Bureau. The multivariate analysis showed that sex was independently associated with *S. mansoni* infection in our study. Consequently, the prevalence of *S. mansoni* was significantly higher in males than females. It was consistent with findings from different parts of Ethiopia [19–22]. However, higher prevalence of *Schistosoma mansoni* infection in females than males was reported in different localities of Ethiopia such as Gorgora [19], Tseda [20], and Zarima [21]. These inconsistent reports might be attributed to differences in water contact behavior of males and females. For instance, males encounter frequent water contacts for swimming, bathing and agricultural activities (irrigation), while females reported more contacts for household activities such as washing clothes and/or utensils and fetching water for household consumption from rivers/streams.

Age was also the other independent variable found to be significantly associated with *S. mansoni* infection in the multivariate analysis. Accordingly, the prevalence of *S. mansoni* was higher in the oldest age group (11–14) years. This is consistent with reports of many researchers in different localities of Ethiopia in which schistosomiasis was consistently higher in the second decade of life [22–25]. This might be justified by the fact children in this age group encounter frequent/regular water contacts for leisure (swimming, bathing) and agricultural activities (irrigation) and washing clothes. Similarly, higher rate of infection was observed for rural children than urban counterparts. This is in agreement with other studies conducted in northwest Ethiopia and North Ethiopia [24].This might be due to the higher tendency of infested water exposure of rural children as they are frequently engaged in water-based activities such as irrigation as they inhabit in the proximity of Abay and Andasa rivers.

The habit of frequent contact with cercariae-infested water such as swimming in the river, washing clothes and utensils using river water, and irrigation activities showed a statistically significant association with *S. mansoni* infection. This is similar to the previous findings reported in Ethiopia [21, 22, 26, 27], and elsewhere [28, 29]. The prevalence of *S. mansoni* was higher in school children who had a habit of frequent swimming, washing clothes, bathing and engaging in irrigation activities than those who did not. This might be due to the presence of cercariae infected water bodies in the surrounding of study areas.

The overall prevalence of hookworm in this study is 41.1%. It was comparatively lower than previous reports from different areas of Ethiopia [30, 31], and Paraguay [32]. Higher than the present finding was reported in Gorgora [19], Mirab Abaya [33], western Ethiopia [34], south Ethiopia [35], Plateau State Nigeria [36, 37], and Thailand [38]. The observed differences in the rate of infection could be due to variations in geography and types of soil, socio-economic conditions, hygienic practices of the population, the methods employed for stool examination and the time of the study, and sample size used.

Several cross-sectional studies have identified the risk factors of hookworm infection in the world. Hookworm infection is acquired via exposure to the soil where filariform larvae live in and penetrate human skin. In addition, *Ancylostoma duodenale* is also acquired by ingestion of food contaminated by the filariform larvae. Similarly, our study showed several factors are significantly related to hookworm infection. Consequently, the odds of hookworm infection was 1.9 times higher in boys than girls. It was in agreement with studies conducted in certain parts of Ethiopia [30, 39, 40] and Brazil [41]. The higher prevalence of hookworm in boys in our study might be because boys usually help their parents in agricultural activities where shoe wearing is not convenient. We also observed that most of the school children and particularly boys wore non-protective shoes (“ergendo” in Amharic).

Multivariate analysis also revealed that children aged 10–14 years were 2.3 times at higher odds of hookworm infection compared to their younger counterparts. This might be attributed to their play behavior and lack/ignorance of shoe wears. On the other hand regular shoe wearing was found to protective for hookworm infection in our study (AOR = 0.3, 95% CI: 0.16–0.50; *P* = 0.00). It was in agreement with other studies conducted in different parts of Ethiopia [21, 42].

This might be because the mode of transmission of hookworm species is often through barefoot penetration by infective filariform larvae.

Eating raw vegetables was also strongly associated with hookworm infection. This might be explained by fact that *Ancylostoma duodenale* can be acquired by ingestion of filariform larvae in food, and this epidemiological feature can possibly occur in areas where *A. duodenale* predominates [43]. However, this study lacks evidence on the hookworm species that prevails in the study area. Rural children were at higher odds of hookworm infection than their urban counterparts. This might be attributed to a higher frequency of exposure to soil` due to lack of shoes, and during agricultural activities, playing and coming to the school of the rural children.

## Conclusion

Hookworm infection and schistosomiasis are public health concerns in the study area. Various demographic characteristics, water based activities and recreational water contact were strongly associated with *S. mansoni* infection. Similarly, failure to wear shoe regularly and other risky behaviors of children, such as geophagy and eating raw vegetables were also predictors of hookworm infection. Hence health education should be delivered regularly to minimize/avoid the risky behaviors and water-based activities and deworming programs should also be implemented on a regular basis.

## Abbreviations

AOR: Adjusted Odds Ratio
CI: Confidence interval
OR: Odds ratio
SPSS: Statistical Product and Service Solutions

## References

[1] WHO (1987). Prevention and control of intestinal parasitic infections, WHO technical report series 741.

[2] WHO. Soil-transmitted-helminth infections. http://www.who.int/en/news-room/fact-sheets/detail/soil-transmitted-helminth-infections.

[3] Crompton DW (1999). How much helminthiasis is there in the world?. J Parasitol.

[4] Ross AG, Bartley PB, Sleigh AC, Olds GR, Li Y, Williams GM, McManus DP (2002). Schistosomiasis. N Engl J Med.

[5] Leykun J (2001). Soil transmitted- helminthic infection and S. Mansoni in school children from Chilga district, Northwest Ethiopia. Ethiop J Health Sci.

[6] Ohaeri CC, Orji NB (2013). Intestinal parasites among undergraduate students of Michael Okpara university of agriculture, Umudike Abia state, Nigeria. World Appl Sci J.

[7] WHO (2002). Prevention and control of schistosomiasis and soil-transmitted helminthiasis.

[8] Obiamuiwe BA (1990). Nmorsi. Human gastro-intestinal parasites in Bendel state, Nigeria. Nigerian J Parasitol.

[9] Nokes C, Cooper ES, Robinson BA, Budy DAP (1991). Geohelminth infection and academic assessment in Jamaican children. Trans R Soc Trop Med Hyg.

[10] Albonico M, Allen H, Chitsulo L, Engels D, Gabrielli AF (2008). Controlling soil-transmitted helminthiasis in pre-school-age children through preventive chemotherapy. PLoS Negl Trop Dis.

[11] Brooker S, Moulin A, Luoba D, Bundy M, Kremer D (2000). Epidemiology of single and multiple species of helminth infections among school children in Busia district. Kenya East Afr Med J.

[12] Lima E, Rocha R, Leite M, Carneiro R, Colley D, Gazzinelli G, Katz N (1991). A multivariate analysis of socio-demographic factors, water contact patterns and Schistosoma mansoni infection in an endemic area in Brazil. Rev Inst Med Trop São Paulo.

[13] Jemaneh L (1998). Schistosoma mansoni and geo-helminthiaisis in school children in Dembia plains. Northwestern Ethiopia Ethiop J Health Sci.

[14] Jemaneh L (1999). Intestinal helminth infection in school children in Gondar town and surrounding areas, Northwest Ethiopia. Ethiop J Health Sci..

[15] Jemaneh L. The epidemiology of Schistosoma mansoni and soil transmitted helminths in elementary school children from the South Gondar zone of the Amhara national regional state, Ethiopia. Ethiop Med J. 2008:105–16. 11144882

[16] Roma B, Worku S (1997). Magnitude of Schistosoma mansoni and intestinal helminthic infections among school children in Wondo genet Zuria. Southern Ethiopia Ethiop J Health Dev.

[17] Mammo B, Asseffa B, Loc T (1989). (). Intestinal helminths in Akaki town, with special emphasis on epidemiology of Schistosoma mansoni. Ethiopian Med J.

[18] Erko B, Tedla S (1993). Intestinal helminths infection in Zeghie, Ethiopia, with emphasis on *Schistosoma mansoni*. Ethiop J Health Dev.

[19] Essa T, Birhane Y, Endris M, Moges A, Moges F (2013). Current status of *Schistosoma mansoni* and associated risk factors among students in Gorgora town, Northwest Ethiopia. International Scholarly Research Notices.

[20] Moges F, Belyhun Y, Tiruneh M, Kebede Y, Mulua A, Kassu A. Intestinal parasite infections in association with cutaneous fungal infection and nutritional status among schoolchildren in Tseda, northwest Ethiopia. Ethiop J Heal Biomed Sci. 2010;3(1):35–43.

[21] Alemu A, Atnafu A, Addis Z, Shiferaw Y, Teklu T, Mathewos B, Birhan W, Gebretsadik S, Gelaw B (2011). Soil transmitted helminthes and Schistosoma mansoni infections among school children in Zarima town, Northwest Ethiopia. BMC Infect Dis.

[22] Awoke W, Bedimo M, Tarekegn M (2013). Prevalence of schistosomiasis and associated risk factors among students attending at elementary schools in Amibera district, Ethiopia. Open J Prev Med.

[23] Grum T (2005). The prevalence of intestinal helminthic infections and associated risk factors among school children in Babile town, eastern Ethiopia. Ethiop J Health Dev.

[24] Assefa A, Dejenie T, Tamass Z (2013). Infection prevalence of Schistosoma mansoniand associated risk factors among schoolchildren in suburbs of Mekelle city, Tigray. North Ethiopia MEJS.

[25] Mitiku H, Legesse M, Teklemariam Z, Erko B (2010). Transmission of Schistosoma mansoni in TikurWuha area. Southern Ethiopia. Ethiop J Health Dev..

[26] Legesse L, Erko B, Hailu A (2010). Current status of intestinal schistosomiasis and soil-transmitted helminthiasis among primary school children in Adwa town. Northern Ethiopia Ethiop J Health Dev.

[27] Endris M, Lemma W, Belyhun Y (2010). Prevalenceof intestinal parasites and associated risk factors among students of Atse fasil general elementary school Azezo, Northwest Ethiopia. Ethiop J Heal Biomed Sci.

[28] Handzel T, Karanja DMS, Addiss DG (2003). Geographic distribution of schistosomiasis and soil-transmitted helminthes in western Kenya: implications for anthelminthic mass treatment. Am J Trop Med Hyg..

[29] Center for infectious disease control report (2008). National prevalence survey on soil-transmitted helminths and schistosomiasis in school children. Rwanda.

[30] Legesse M, Erko B. Prevalence of intestinal parasites among schoolchildren in a rural area close to the southeast of Lake Langano. Ethiopia Ethiop J Health Dev. 2004;18(2)

[31] Merid Y, Hegazy M, Mekete G, Teklemariam S. Intestinal helminthic infection among children at Lake Awassa area, South Ethiopia. Ethiop J Health Dev. 2001;15:31–7:57–78.

[32] Labiano-Abello N, Canese J, Velazquez ME, Hawdon JM, Wilson ML, Hotez PJ (1999). Epidemiology of hookworm infection in Itagua, Paraguay: a cross sectional study. Mem Inst Oswaldo Cruz.

[33] Ketema H, Biruksew A, Mekonnen Z (2015). Prevalence of Necator americanus infection and risk factors among school-age children in Mirab Abaya district, South Ethiopia. Asian Pac J Trop Dis.

[34] Samue F, Demsew A, Alem Y, Hailesilassie Y (2017). Soil transmitted helminthiasis and associated risk factors among elementary school children in ambo town, western Ethiopia. BMC Public Health.

[35] Erosie L, Merid Y, Ashiko A, Ayine M, Balihu A, Muzeyin S, Teklemariam S, Sorsa S (2002). Prevalence of hookworm infection and hemoglobin status among rural elementary school children in southern Ethiopia. Ethiop J Health Dev.

[36] Bala AY, Yakubu DP (2010). A survey of hookworm infection among pupils of school age in Jos-north, plateau state, Nigeria. Nig J Basic Appl Sci.

[37] Odebunmi JF, Adefioye OA, Adeyeba OA (2007). Hookworm infection among school children in Vom, plateau state. Nigeria Eur J Sci Res.

[38] Jiraanankul V, Aphijirawat W, Mungthin M, Khositnithikul R, Rangsin R, Traub RJ, Piyaraj P, Naaglor T, Taamasri P, Leelayoova S (2011). Incidence and risk factors of hookworm infection in a rural community of Central Thailand. Am J Trop Med Hyg.

[39] Girum T (2015). Prevalence of intestinal parasitic infections among patients with diarrhea at Wonago health center, southern Ethiopia: a retrospective study. JIID.

[40] Asrat A, Tewodros D, Alemayehu W (2011). Prevalence and risk factors of IPs among Delgi school children, northern Gonder, Ethiopia. J Parasitol Vector Biol.

[41] Santos FLN, Souza AMGC, Soares NM (2013). Hookworm and threadworm infections and their association with hemoglobin and eosinophil concentrations in residents of Salvador Bahia, Brazil. Rev Inst Med Trop São Paulo.

[42] Abera B, Alem G, Yimer M, Herrador Z (2013). Epidemiology of soil-transmitted helminths, Schistosoma mansoni, and haematocrit values among schoolchildren in Ethiopia. J Infect Dev Ctries.

[43] Gillespie SH, Pearson RD (2001). Principles and practice of clinical parasitology.

